# Habitude alimentaire des mères pendant la grossesse et l’allaitement, région Amoron’i Mania Madagascar: étude qualitative

**DOI:** 10.11604/pamj.2018.29.194.12873

**Published:** 2018-04-02

**Authors:** Lantonirina Ravaoarisoa, Julio Rakotonirina, Daniel Andriamiandrisoa, Perrine Humblet, Jean de Dieu Marie Rakotomanga

**Affiliations:** 1 Institut National de Santé Publique et Communautaire, Mahamasina, Antananarivo, Madagascar; 2 Faculté de Droit, d’Economie, de Gestion et de Sociologie, Université d’Antananarivo, Madagascar; 3 Ecole de Santé Publique, Université Libre de Bruxelles, Belgique

**Keywords:** Dietary habits, pregnancy, breastfeeding, diet, Habitude alimentaire, grossesse, allaitement, alimentation

## Abstract

**Introduction:**

L'alimentation des mères est très importante à cause des besoins nutritionnels spécifiques pour la reproduction. La présente étude vise à décrire les habitudes alimentaires des mères pendant la grossesse et l'allaitement et répertorier les facteurs influençant ces habitudes.

**Méthodes:**

Une étude qualitative a été menée auprès des femmes enceintes et allaitantes vivant dans la région Amoron'i Mania, Madagascar. Huit focus group (6 à 10 femmes par groupe) et 23 entretiens individuels ont été réalisés pour collecter les données. L'analyse thématique a été utilisée et a porté sur la description des habitudes alimentaires à partir de la conduite de l'alimentation des femmes et sur les déterminants socioculturels et économiques des habitudes décrites.

**Résultats:**

Pendant la période de grossesse et d'allaitement, l'habitude alimentaire des femmes ne varie pas considérablement exceptée au tout début de l'allaitement. Elles ont une alimentation faiblement diversifiée et monotone, pauvres en fruits et légumes et pauvre en protéines. C'est au tout début de l'allaitement, pendant la pratique de la tradition appelée « mifana », que les femmes bénéficient d'une alimentation plus riche que d'habitude. Ces habitudes alimentaires sont influencées par le type de productions agricoles de la région et leur disponibilité dans l'année (autoconsommation), le pouvoir d'achat (en cas de pénurie) ainsi que la tradition.

**Conclusion:**

L'habitude alimentaire des mères semble inadéquate. Approfondir les connaissances sur les déterminants de leur pratique alimentaire est nécessaire.

## Abstract (en)

**Introduction:**

Women's dietary habits are very important due to the specific nutritional requirements for reproduction. This study aimed to describe women's dietary habits during pregnancy and breastfeeding and to identify factors influencing these habits.

**Methods:**

We conducted a qualitative study of pregnant and breastfeeding women living in Amoron'i Mania region, Madagascar. Eight focus groups (6-10 women per group) and 23 individual interviews were carried out to collect data. Thematic analysis was used and focused on the description of women's dietary habits on the basis of dietary behaviours as well as of sociocultural and economic determinants of the described habits.

**Results:**

During pregnancy and breastfeeding, women's dietary habits did not vary considerably except at the very beginning of breastfeeding. They had a little diversified and monotonous diet, poor in fruit and vegetables and poor in proteins. At the very beginning of breastfeeding, during the practice of “mifana ” tradition, women had a diet more rich than usual. These dietary habits were influenced by the type of agricultural products in the region and by their availability during the year (self-consumption)as well as by purchasing power (in case of shortage) and tradition.

**Conclusion:**

Mothers' dietary habits appear to be inadequate. This study highlights the importance of improving knowledge of the determinants of mothers' dietary behaviours.

## Introduction

La reproduction engendre chez les mères des besoins nutritionnels spécifiques pour assurer la croissance, le développement, la santé et les différents soins de leurs progénitures [1]. Les mères constituent ainsi un groupe vulnéra' l'enfant, comme le retard de croissance intra-utérine, le faible poids à la naissance, la malnutrition infantile et l'hémorragie autour de la naissance; tous ces effets néfastes concourent à un risque élevé de mortalité maternelle et infantile [2]. L'alimentation et l'état de santé sont les principaux déterminants de l'état nutritionnel. Ils dépendent de plusieurs facteurs d'ordre économique, social, culturel, environnemental, etc. Concernant les mères dans les pays à faible revenu, les caractéristiques liées à leurs parcours de reproduction tels que la parité et l'intervalle inter génésique s'ajoutent à ces facteurs [3]. Ainsi, l'alimentation d'une mère pendant son parcours de reproduction, essentiellement pendant la grossesse et l'allaitement, est très importante. La malnutrition maternelle est une préoccupation mondiale. La proportion de femmes qui souffrent de dénutrition (Indice de Masse Corporelle ou IMC < 18,5) se situe au-delà de 20% pour de nombreux pays à faibles et moyennes ressources et elle va même jusqu'à 40% pour certains [4]. Madagascar a enregistré une prévalence élevée de dénutrition (IMC < 18.5) chez les femmes en âge de procréer (26,7% en 2008-2009) [5]. Des connaissances approfondies sur les causes de la dénutrition maternelle s'avèrent utiles pour mener à bien la lutte. La présente étude a pour objectif de décrire les habitudes alimentaires des mères au cours de leur parcours de reproduction et de répertorier les facteurs socioculturels et économiques influençant sur ces habitudes.

## Méthodes

La présente recherche qualitative a été conduite en février et mars 2014 dans la région Amoron'i Mania Madagascar. Cette région détient la prévalence la plus élevée de dénutrition (IMC < 18,5) chez les femmes en âge de procréer à Madagascar avec 41,6% en 2008-2009 [5].

**Site de l'étude**: La région Amoron'i Mania est une des 22 régions qui forment la grande île de Madagascar. Elle est composée de 4 districts sanitaires, 53 communes dont 52 sont rurales et compte 580 000 habitants environs. L'étude s'est déroulée en zone rurale en raison de l'importance du problème de malnutrition dans ce milieu par rapport au milieu urbain [**5**]. La Région se trouve dans les hautes terres centrales du pays et habitée principalement par l'ethnie appelée « Betsileo ». L'agriculture constitue la principale activité de la population dont essentiellement l'agriculture de subsistance [6].

**Collecte des données**: Une commune par district a été choisie pour l'enquête. Le choix a été fait de manière raisonnée en veillant à ce que les 4 communes représentent mieux leur district mais intègrent la diversité au sein de la région. Vingt-trois entretiens individuels et 8 entretiens de groupe ont été réalisés auprès des femmes enceintes et allaitantes. L'entretien individuel s'est adressé à dix femmes enceintes et treize femmes allaitantes. Les personnes interviewées ont été recrutées dans des villages choisis aléatoirement dans chacune des 4 communes et avec l'aide des agents communautaires locaux et d'autres personnes ressources tels les membres des villages. Le nombre de personnes enquêtées a été déterminé en fonction de la saturation des informations. Environ 5 à 6 entretiens par commune ont été conduits. Ils s'agissaient d'entretiens semi-directifs qui utilisaient comme base d'entretien le même guide que l'entretien de groupe. Les entretiens individuels représentaient une source complémentaire aux entretiens en groupe focal, renforçant les résultats de ces derniers ou permettant de mettre en évidence des changements par rapport à la tradition. Pour la discussion en groupe focal, deux réunions par commune ont eu lieu, une pour le groupe de femmes enceintes et une autre pour les femmes allaitantes. Chaque groupe est composé de 6 à 10 femmes. L'entretien s'est tenu dans un local du Centre de Santé de Base (CSB) de la commune. Les groupes ont été formés de femmes enceintes venues pour les séances de consultation prénatale ou de femmes allaitantes amenant leur enfant pour la séance de vaccination dans les CSB. Les participants ont été rassemblés avec la collaboration du personnel de santé. Les discussions en groupe ont porté sur les opinions, les points de vue collectifs au sujet de l'alimentation des femmes enceintes et/ou allaitantes ainsi que sur la tradition, les interdits et recommandations alimentaires, les rites et rituels relatifs aux périodes de la grossesse et post-natale. Elles s'appuyaient sur l'utilisation d'un guide d'entretien. Le guide d'entretien a été élaboré suite à une étude documentaire qui consiste à cerner le sujet dans son ensemble et à dégager les points à approfondir et il a été pré-testé. L'étude a obtenu l'accord de la Comité d'Ethique du Ministère de la Santé Publique de Madagascar.

**Analyse des données**: Après codage et regroupement des résultats de chaque entretien, l'analyse thématique a porté sur la description des habitudes alimentaires à partir de la conduite de l'alimentation de la femme enceinte et allaitante et sur les déterminants socioculturels et économiques des habitudes décrites.

## Résultats

### Description des habitudes alimentaires

**Alimentation des femmes enceintes**: Les femmes enceintes prennent le repas trois fois par jour. Toutes les données recueillies ont mis l'accent sur l'omniprésence du riz dans l'alimentation. Celui-ci représente le plat principal, les femmes en consomment au moins deux fois par jour: le matin, le midi et le soir ou deux d'entre eux seulement. En période de soudure, certaines femmes prennent le riz seulement le soir. Le riz est habituellement accompagné d'autres aliments de supplément appelée « laoka ». Ce dernier est constitué de feuilles vertes de type saisonnier le plus fréquemment, de légumineuses genre haricot et « voanjobory » le moins souvent et rarement de la viande. L'une des participantes a déclaré en entretien: *« comme plat de base, je mange quotidiennement du riz trois fois par jour, le matin, le midi et le soir. Le riz est souvent accompagné de légumes du type feuilles vertes, des « voanjobory » ou d'autres types de “voamaina ” (légumineuse) et parfois de la viande (...) »*. Les légumes figurent parmi les accompagnements du riz mais les participantes en consomment moins souvent par manque de moyen pour s'en procurer au marché. En discussions de groupe, toutes les femmes adhèrent à l'affirmation que la fréquence de consommation du riz et de la viande et des légumes dépend du niveau de vie de la famille et de la saison. La famille aisée consomme du riz trois fois par jour et de la viande une ou deux fois par semaine. En période de soudure, certaines femmes des familles moins aisées prennent le riz seulement le soir et la viande moins d'une fois par mois. Le riz est remplacé par des tubercules (patate douce et manioc) et quelque fois par du maïs. Ces substituts sont consommés sans accompagnement. Selon le récit d'une femme enceinte: *« (...). Le riz s'est parfois substitué par du manioc, des patates douces, du maïs. Comme notre production rizicole n'arrive pas à couvrir toute l'année, les tubercules sont notre solution »*. La substitution du riz deviendra récurrente voire une habitude quand la production rizicole est insuffisante. Certains produits de cueillette tels les fruits sont suffisamment rares pour que les mères n'en tirent pas profit: *« Nous mangeons peu de fruits. Saisonniers, ils sont presque non disponibles: quand la saison des oranges arrive, nous mangeons deux ou trois et c'est fini. Il en est de même pour les pommes et les pêches »*.

D'une manière générale, l'alimentation des femmes ne connait pas de variation considérable dans la semaine. Certaines femmes disent qu'elles achètent de la viande ou des légumes le jour du marché quand elles ont le moyen d'en acheter. Selon le récit d'une femme au cours d'une discussion de groupe: *« Je ne vois pas de changement sur notre alimentation même le samedi et dimanche. Quelque fois, nous achetons de la viande ou des poissons séchés au marché quand nous avons de l'argent »*. Toutes les femmes affirment qu'elles consomment toujours de la viande durant les jours de fête. Une variation saisonnière de l'alimentation est remarquée. Les feuilles vertes sont disponibles toute l'année mais le type change en fonction de la saison. Les femmes racontent aussi qu'elles peuvent cueillir des poissons frais dans les rizières après la récolte du riz et elles en mangent plus souvent pendant cette période. Les femmes enceintes ne suivent pas de régime particulier et s'associent au repas familial. Selon le propos d'une femme : *« (...) Mon régime alimentaire ne change pas si on le compare à celui que j'ai mangé avant la grossesse. Il ne diffère pas de celui des autres membres de ma famille »*. La connaissance de la valeur nutritive et hygiénique des aliments a été mise en évidence chez les participantes. Les besoins en énergie, en protéine et en vitamines ainsi que les aliments riches en ces éléments sont bien connus et évoqués pendant les discussions de groupe. *« (...) Le régime alimentaire pendant la grossesse exige des aliments riches en vitamines comme les légumes, les fruits et une grande quantité de riz; d'une meilleure nourriture telle que la viande ou des aliments bons pour la santé. Puisque certains aliments comme les légumineuses sont souvent difficiles à digérer »*. La consommation de ces aliments riches n'a pas été mise en évidence dans la pratique alimentaire des femmes.

**Alimentation de la femme allaitante**: L'alimentation de la femme allaitante fait l'objet d'un changement explicite juste après l'accouchement, particulièrement pendant la *période de réclusion post-natale: « mifana »*(être mise au chaud). Pendant cette période qui dure un à trois mois environ, le riz s'accompagne très souvent des mets spéciaux tels que la viande et les cucurbitacées. On lui prépare également un plat rituel le *« ropatsa »*(bouillons de petites crevettes) connu comme favorisant la montée laiteuse. Une mère allaitante déclare: *« Pendant que je me suis mis au chaud “mifàna ” après l'accouchement, j'ai suivi un régime particulier durant environ un mois, j'ai mangé successivement de la viande de boeuf, de porc et de poulet. (...) J'absorbe de temps en temps du "ropatsa" (bouillon de petites crevettes) et des cucurbitacées avec du riz, favorables à la montée du lait »*. Après cette période de réclusion post-natale *« mifana »*, les femmes allaitantes reprennent le rythme ordinaire de la vie quotidienne. Elles s'associent aux repas familiaux et ne bénéficient plus de traitement spécifique en matière d'alimentation. C'est ce qui est évoquée au cours des discussions de groupes: *« Dès que cette période de « mifana » s'est terminée, la femme commençais à prendre des repas communs à tous les membres de la famille »*. Cependant, certaines femmes ont évoqué l'évitement de certains aliments tels que les tubercules, les feuilles vertes et les légumineuses ou la réduction de leur consommation pendant les premiers mois d'allaitement. Elles avancent que ces aliments détériorent la qualité du lait maternel. La consommation de certains aliments comme le lait est aussi évoquée. *« Je m'abstiens encore du manioc et des patates douces pendant environ 5 mois après l'accouchement. Puis, un changement a lieu au niveau de l'alimentation, à savoir prendre une bonne quantité de riz et, entre-temps, du pain avec du lait. Alors que je n'en ai pas cette habitude avant l'accouchement. (...) ». « Au cours de l'allaitement, je mange moins d'haricots, de voanjobory (légumineuse), de voanemba (légumineuse) et du manioc, des patates douces, du maïs ou du taro, alors que je m'efforce de boire du lait tous les matins. Pourtant la quantité des aliments reste constante »*.

### Déterminants des comportements alimentaires

### Période de grossesse

**Hygiène de l'alimentation et interdits coutumiers alimentaires**: Les femmes enceintes se voient encore observer certains interdits alimentaires traditionnels de la famille ou religieux, comme certains types de viande proscrits (porc, mouton, hérisson). Traditionnellement, les *« hanikotrana »* (tubercules) tels le manioc, les patates douces, les taros et les aliments riches en graisse sont aussi à déconseiller: *«Concernant l'alimentation, je ne mange pas des "hanikotrana" et des aliments riches en graisse. Ils sont déconseillés pour éviter l'accouchement difficile»* . Cependant, ces comportements alimentaires traditionnels s'estompent progressivement chez certains groupes. Chez les femmes ayant un certain niveau d'instruction, les informations reçues auprès des agents de santé au cours de la consultation prénatale sont la raison évoquée: *« Nous n'avons pas encore entendu parler des interdits alimentaires propres à la femme enceinte. De même, les arachides riches en matière grasse n'ont aucun effet sur la grossesse selon le médecin »*. Mais le problème d'accès aux aliments déjà en pénurie constitue aussi une raison de non suivi des interdits. Au cours des discussions de groupe, la majorité des femmes enceintes sont en accord avec ce récit: *« Il existe en principe des interdits auxquels les gens devraient se soumettre, mais personne ne les observe à présent. Quant à nous, nous ne nous privons rien puisqu'ici, il est difficile de trouver de quoi manger »*. A côté des aliments interdits existe quelques aliments recommandés. La femme enceinte est tenue de suivre un régime alimentaire particulier dont le but est de lui assurer un accouchement facile et de pourvoir l'enfant de la santé, la force et l'intelligence. Elles reconnaissent que la viande, les poissons, les œufs sont bons pour les femmes enceintes. On leur recommande aussi de manger des aliments riches en vitamines comme le lait, les feuilles vertes riches en fer (les cressons, feuilles de patate douce) et les fruits de toutes sortes. Ces recommandations proviennent essentiellement des agents de santé lors des séances de consultations prénatales et des agents communautaires qui travaillent pour la santé.

**Période post-natale**: *Soins portés à la mère pendant qu'elle se met au chaud « mifàna »*: après l'accouchement, la femme et le nouveau-né doivent s'installer dans un endroit bien préparé par la famille pour se mettre au chaud « mifana ». Inactive, elle se repose au lit, pendant un ou deux mois environ, suivant la situation économique de la famille, en utilisant des couvertures chaudes. Pendant cette période, le repas de la mère est servi au lit et tous ses plats ont la vertu de favoriser la montée laiteuse et la production du lait et aident la femme en couche à récupérer progressivement ses forces. *« Après l'accouchement, je me suis mise au chaud pendant un mois, c'est-à-dire je me suis reposée au lit. J'ai suivi un régime alimentaire spécial qui relève de la bonne qualité. Je mange ce qui paraît bien pour le lait du nouveau-né »*.

**Rituel du « ropatsa » ou bouillon de petites crevettes**: Les cadeaux offerts lors de la naissance d'un enfant prennent le nom générique de « ropatsa » (bouillons de crevettes). Actuellement, ils se présentent sous forme d'argent ou en nature (viande, volailles, riz) étiquetés toujours *« ropatsa »*. Comme l'a confirmé une jeune femme: *« Les gens pratiquent encore le « ropatsa » et il se fait généralement en argent variant de 500 Ariaryà 1.000 Ariary (de 0,20 à 0,40 USD). Il arrive parfois des gens qui offrent beaucoup plus. Ça dépend des relations avec le visiteur ». Une autre y ajoute: « Les gens pratiquent encore le rituel de "ropatsa", certains ont donné un "daba" de paddy (= 12kg) ou de l'argent (5.000 Ar), d'autres ont offert des aliments d'accompagnement du riz comme une poule »*.

### Contraintes économiques

**Pénurie alimentaire et insuffisance du pouvoir d'achat**: La région connait une pénurie alimentaire pendant la période de soudure bien connue sous le nom de *« fahavaratra »*. Les mères ont évoqué comme raison l'insuffisance des productions agricoles qui n'arrive pas à couvrir l'année entière. En général, la population cultive pour l'autoconsommation. *« Nous ne vendons pas nos produits agricoles, nous les utilisons seulement comme nourriture »* . En période de récolte, elle en vend juste une partie pour pouvoir se procurer des produits de première nécessité: huile, pétrole, sucre, sel, etc. Pendant la période de soudure, mari et femme doivent s'adonner à des travaux de salariat agricole *« sarakantsaha »* pour pouvoir acheter des aliments et d'autres biens. L'insuffisance du pouvoir d'achat des paysans se traduit par le comportement alimentaire de la femme enceinte et allaitante: *« Ici je ne peux pas me permettre de consommer tous les aliments appropriés à la femme enceinte, puisque l'argent a fait défaut. Je ne crois pas être en mal de nourriture, c'est le manque d'argent qui fait souffrir le plus »*. Les produits de l'élevage ne sont pas destinés à l'alimentation. Ils sont vendus pour l'achat des besoins un peu plus importants (vêtement, couverture, fournitures scolaires) ou pour des besoins urgents (soins et médicaments). C'est une sorte de réserve qui permet à la population rurale de générer de l'argent: *« Nous mangeons rarement des volailles, seulement quand il y a une malade dans la famille ou à l'occasion d'une fête, fête nationale, Noël, nouvel an ou quand nous avons des visiteurs comme mes beaux-parents. Nous les vendons quand nous avons besoin d'argent »*.

## Discussion

**Alimentation des femmes enceintes**: La description de la consommation des femmes enceintes reflète celle de leur ménage. Elle vertes ou des tubercules), pauvres en fruits et légumes et pauvre en protéine (légumineuse quelquefois et viande rarement). C'est une pratique alimentaire basée sur la tradition ainsi que sur les aliments disponibles dans la région. Le riz constitue l'aliment de base des Malagasy et il s'inscrit dans l'univers culturel de l'ethnie Betsileo, habitant cette région [7]. La culture de manioc et celle de patate douce, aliments de substitution du riz, se trouvent respectivement en deuxième et en troisième place après la culture du riz [8]. La viande, rarement consommée, pourrait provenir de l'élevage qui n'est pas destiné à la consommation mais à la vente pour assurer les besoins autres que la nourriture. La corrélation entre le type de production et les aliments consommés est importante dans une population qui vit de l'autoconsommation [9]. D'une manière générale, l'alimentation des femmes ne subit pas de modification pendant la grossesse. Les recommandations des agents de santé sur la consommation des aliments riches en protéines et en vitamines ne se traduisent pas en pratique. Dans la tradition Malagasy, la femme enceinte est incitée à prendre soins de leur alimentation et de ne pas manger trop pour éviter un accouchement difficile secondaire au gros bébé [10]. Selon un proverbe Malagasy:*« une femme enceinte qui mâche tout ce qu'elle trouve: elle aura un jour difficile à passer, elle enfantera douloureusement »* [11]. Selon les études sur les pratiques alimentaires pendant la grossesse, la restriction alimentaire est fréquemment observée et la peur d'avoir un gros bébé qui rendrait difficile l'accouchement constitue la principale raison évoquée [12-14].

**Alimentation des femmes allaitantes**: L'alimentation des femmes change complètement pendant la période de réclusion post-natale appelée « mifana ». La consommation des aliments riches est constatée. Tous les plats offerts aux femmesen post-partum auraient la vertu de favoriser la montée laiteuse et la production du lait et d'aider la femme en couche à récupérer progressivement ses forces. L'amélioration de l'alimentation des femmes à cette phase de début de l'allaitement est connue dans beaucoup de pays. Des changements en termes de quantité et qualité selon le pays sont observés, mais la consommation des aliments chauds est généralement recommandée [15-17]. Après cette phase de réclusion, l'alimentation de la mère allaitante redevient comme avant. Toutefois, chez certaines femmes, la privation de certains aliments est signalée, tels que les feuilles vertes et les tubercules jugés mauvais pour la qualité du lait maternel aux premiers mois de l'allaitement. Le choix des aliments à conseiller ou non pendant l'allaitement dépend de leur classification en aliment chaud ou froid et chaque pays a la définition de ce qu'il appelle aliment froid ou chaud [12]. Généralement, la consommation des aliments froids est déconseillée pendant l'allaitement car ils sont considérés comme non favorable à la santé des femmes allaitantes et affectent négativement la quantité et la qualité de la production de lait [15].

**Interdits alimentaires**: Selon certaines femmes enceintes, certains aliments sont interdits durant la grossesse. C'est le cas de l'arachide, aliment riche en graisse, qui est relatée comme favorisant l'accouchement d'un gros bébé. Ce que les ancêtres ont appris au sujet des interdits alimentaires demeure au cours des générations. Mais certaines ne les respectent plus. Selon la présente étude, des femmes ayant un certain niveau d'instruction affirment qu'elles ne tiennent plus compte de ces interdictions. Une étude menée au Ghana montre que les femmes enceintes plus instruites adhèrent moins à ces interdictions [18]. Elles peuvent être plus réceptives aux conseils et aux recommandations des agents de santé et rejettent facilement les interdictions alimentaires sans fondement. Chaque pays a ses propres interdictions en matière d'alimentation des femmes enceintes et allaitantes [14, 19]. Ce sont les impacts de ces interdictions sur l'état nutritionnel et sanitaire des femmes qui s'avèrent intéressants et méritent d'être évalués.

**Tradition « mifana »**: Pendant la phase de réclusion après l'accouchement appelée « mifana », les femmes bénéficient d'un repos et d'une alimentation plus riche que d'habitude. Ces conditions sont favorables à un bon état nutritionnel. C'est une sorte de congé de maternité de la société traditionnelle Malagasy. Actuellement, la durée de cette phase varie d'une femme à l'autre et selon la possibilité de la famille [20]. Le cadeau offert après l'accouchement appelé « ropatsa » peut aider la famille. Ce genre de coutume qui accorde un moment de repos aux femmes accouchées existe dans plusieurs régions du monde mais avec des spécificités. Dans tous les cas, le bien-être du nouveau-né et la récupération de la mère après la grossesse et l'accouchement constituent les objectifs [21, 22].

**Facteurs économiques**: L'environnement économique de la région dans laquelle se situe l'étude limite l'accès des femmes à une bonne alimentation. La pénurie alimentaire due à l'insuffisance des productions agricoles et la limitation du pouvoir d'achat sont rapportées par les femmes. La situation semble plus difficile en période de soudure. Les femmes connaissent la valeur nutritive et hygiénique des aliments mais la priorité c'est de trouver de quoi manger sans se soucier de la qualité. Amoron'i Mania figure parmi les régions les plus pauvres du pays et cette situation expose sa population à un état d'insécurité alimentaire [23]. Cette sécurité alimentaire de la population constitue un déterminant principal de sa pratique en matière d'alimentation [24]. Cette étude est une première étape d'un processus visant à analyser la situation nutritionnelle des mères dans la région Amoron'i Mania. Les résultats obtenus permettront d'orienter la conduite des prochaines étapes et susciteront une analyse plus approfondie d'autres éléments qui aidera à mieux comprendre la problématique de la malnutrition. L'absence des études menées dans d'autres régions de l'île limite la possibilité de comparer les résultats afin de dégager la spécificité de la région. Toutefois, les résultats révèlent des éléments qui pourraient expliquer la malnutrition de la région comme la pratique alimentaire inadéquate de la population et l'insécurité alimentaire surtout en période de soudure.

## Conclusion

L'habitude alimentaire des mères ne subit pas une variation importante au cours de leur parcours de reproduction. Pendant la grossesse, la ration alimentaire n'affiche pas de modification particulière malgré une meilleure connaissance de la valeur nutritive et hygiénique des aliments. Le tout début de la période d'allaitement connait un changement significatif de l'alimentation à cause de la tradition « mifana ». D'une manière générale, l'alimentation des mères semble inadéquate et approfondir les connaissances sur les déterminants de leur pratique alimentaire est nécessaire.

### Etat des connaissances actuelle sur le sujet

- La pratique alimentaire de la mère pendant la grossesse et l'allaitement est une condition sine qua non du développement et de la croissance de ses enfants durant les 500 premiers jours de leur vie;
- Chaque pays à ses habitudes et ces traditions concernant l'alimentation des femmes pendant la grossesse et l'allaitement;
- Dans les pays à faibles ressources où le problème de malnutrition est important, l'insécurité alimentaire est le principal déterminant de l'habitude alimentaire de la population.

### Contribution de notre étude à la connaissance

- Dans la région étudiée, l'habitude alimentaire des mères ne leur permet pas d'avoir une alimentation adéquate;
- Il existe des pratiques traditionnelles qui favorisent une bonne alimentation des mères pendant leur parcours de reproduction;
- L'effet période (pénurie alimentaire de la période de soudure) est un facteur essentiel qui limite l'accès des mères aux aliments dans la région étudiée.

## Conflits d’intérêts

Les auteurs ne déclarent aucun conflit d'intérêts.

## References

[1] Picciano MF (2003). Pregnancy and lactation: physiological adjustments, nutritional requirements and the role of dietary supplements. J Nutr..

[2] Ramakrishnan U, Grant F, Goldenberg T, Zongrone A, Martorell R (2012). Effect of women's nutrition before and during early pregnancy on maternal and infant outcomes: a systematic review. Paediatr Perinat Epidemiol..

[3] Lartey A (2008). Maternal and child nutrition in Sub-Saharan Africa: challenges and interventions. Proc Nutr Soc..

[4] Black RE, Allen LH, Bhutta ZA, Caulfield LE, de Onis M, Ezzati M (2008). Maternal and child undernutrition: global and regional exposures and health consequences. Lancet..

[5] Instat et ICF Marco (2010). Enquête démographique et de santé Madagascar 2008-2009.

[6] Instat (2012-2013). Enquête Nationale sur le suivi des objectifs de Millénaire pour le Développement à Madagascar.

[7] Gendarme R (1963). L'économie de Madagascar: diagnostic et perspectives de développement.

[8] Instat (2011). Enquête périodique auprès des ménages 2010.

[9] Pellegrini L, Tasciotti L (2014). Crop diversification, dietary diversity and agricultural income: empirical evidence from eight developing countries. Canadian Journal of Development Studies / Revue canadienne d'études de développement..

[10] Decary R (1930). Quelques pratiques malgaches relatives aux accouchements. Bulletins et Mémoires de la société d'anthropologie de Paris, VIII° Série. Tome 1 fascicule..

[11] Veyrières P, Méritens G (1967). Le livre de la sagesse Malgache: proverbes, dictons, expressions figures et curieuses Paris. Editions maritimes et d'outre-mer..

[12] Nag M (1994). Beliefs and Practices about Food during Pregnancy. Implications for Maternal Nutrition Economic and Political Weekly..

[13] Nejimu BZ (2015). Food taboos and misconceptions among pregnant women of Shashemene District, Ethiopia, 2012. Science Journal of Public Health Nutr..

[14] Alonso EB (2017). The impact of culture, religion and traditional knowledge on food and nutrition security in developing countries. Foodsecure Working paper..

[15] Liu YQ, Petrini M, Maloni JA (2014). “Doing the month”: postpartum practices in Chinese women. Nurs Health Sci..

[16] Chen LW, Low YL, Fok D, Han WM, Chong YS, Gluckman P (2014). Dietary changes during pregnancy and the postpartum period in Singaporean Chinese, Malay and Indian women: the GUSTO birth cohort study. Public Health Nutr..

[17] Dennis CL, Fung K, Grigoriadis S, Robinson GE, Romans S, Ross L (2007). Traditional postpartum practices and rituals: a qualitative systematic review. Women's health (London, England)..

[18] Otoo P, Habib H, Ankomah A (2015). Food prohibitions and other traditional practices in pregnancy: a qualitative study in western region of Ghana. Advanced in reproductive sciences..

[19] Pelto GH (1987). Cultural issues in maternal and child health and nutrition. Soc Sci Med..

[20] Ravaoarisoa L, Mamelontsoa RJ, Rakotonirina EJ, Randrianarimanana VD, Rakotomanga JDM (2016). “Mifana”: pratique traditionnelle après accouchement et état nutritionnel des mères à Madagascar. Med Afr Noire..

[21] Kim-Godwin YS (2003). Postpartum beliefs and practices among non-Western cultures. MCN Am J Matern Child Nurs..

[22] Aubaille-Sallenave F (1997). Les nourritures de l'accouchée dans le monde arabo-musulman méditerranéen. Médiévales..

[23] Mayen AL, Marques-Vidal P, Paccaud F, Bovet P, Stringhini S (2014). Socioeconomic determinants of dietary patterns in low- and middle-income countries: a systematic review. Am J Clin Nutr..

[24] FAO (1997). Agriculture food and nutrition for Africa.

